# The Ideal Size of Mesh for Open Inguinal Hernia Repair: A Morphometric Study in Patients with Inguinal Hernia

**DOI:** 10.7759/cureus.2573

**Published:** 2018-05-03

**Authors:** Balaiya Anitha, Karuppusamy Aravindhan, Sathasivam Sureshkumar, Manwar S Ali, Chellappa Vijayakumar, Chinnakali Palanivel

**Affiliations:** 1 Anatomy, Jawaharlal Institute of Postgraduate Medical Education and Research (JIPMER), Puducherry, India.; 2 Surgery, Jawaharlal Institute of Postgraduate Medical Education and Research (JIPMER), Puducherry, India.; 3 Surgery, AIIMS, Bhubaneswar, India; 4 Preventive Medicine, Jawaharlal Institute of Postgraduate Medical Education and Research (JIPMER), Puducherry, India.

**Keywords:** lichtenstein’s mesh repair, hernia recurrence, inguinal canal, anterior superior iliac spine, inguinal ligament

## Abstract

Introduction

This study was done to analyze the morphometric features of the inguinal canal with different types of inguinal hernias to determine the appropriate size of mesh required to cover potential sites of recurrence. A morphometric assessment in the particular population is essential to recommend the appropriate mesh size in inguinal hernias to cover all the potential sites of recurrence.

Materials and methods

This was a prospective observational study, including all consecutive patients undergoing open inguinal hernia repair under local/regional/general anesthesia over a period of three years. Surgeries that were done in emergencies for complicated hernias, laparoscopic repair, and recurrent inguinal hernias were excluded. Intra-operative parameters were studied to predict the appropriate mesh size, which included the position of the superficial and deep inguinal ring (SIR and DIR) with the diameter, the distance of SIR and DIR from the anterior superior iliac spine (ASIS), and the distance from the summit of the muscular arch to the inguinal ligament. The differences in morphometric details between the types of hernias and categorical variables were assessed using the chi-square test.

Results

The study included a total of 170 patients with a mean age of 50.67 + 17.59 years. An indirect hernia was the most common type in patients less than 60 years. The mean distance from ASIS to SIR was 10.2+ 1.9 cm, and in indirect hernia patients, it was found to be significantly increased (p=0.042). The mean distance from ASIS to DIR was 4.14+1.57 cm, where the indirect hernia patients had a significantly less distance (p=0.029). The mean length of the inguinal canal in a direct hernia was 5.66 + 0.5 cm, whereas, in an indirect inguinal hernia, it was 6.46 + 0.8 cm, which was significant (p=0.029). The mean distance from the midpoint of the inguinal ligament to the summit of the muscular arch was 4.03 cm, and there was no significant difference between the indirect and direct hernia patients.

Conclusion

After considering the morphometric assessments of the length of the inguinal canal, the mean distance from the midpoint of the inguinal ligament to the summit of the muscular arch, the mean distance from ASIS to DIR, the ideal mesh size for the population would be 9 X 15 cm to cover all the potential sites of recurrence.

## Introduction

The inguinal region is a potential site for the development of an inguinal hernia [1]. Chronic physical straining, which increases the intra-abdominal pressure, acts as a promoting factor for the development of inguinal hernias, which, in turn, alters the inguinal canal anatomy [2]. The length of the inguinal canal varies according to the race, stature, and gender of the person [3]. The morphometric assessment of the inguinal region with respect to inguinal hernias is of essence in the background of mesh repair. The anatomical repair of an inguinal hernia, including Bassini’s repair and Shouldice’s repair, was largely abandoned a decade back due to the high incidence of recurrence, as these techniques did not address the important principle for the prevention of recurrence; that is, the tension-free repair of the posterior wall of the inguinal canal [4].

The current standard for the surgical management of an inguinal hernia that is adopted in a majority of the centers around the world is Lichtenstein’s tension-free mesh repair of the posterior wall inguinal canal [5]. The posterior wall is strengthened by the formation of a fibrous frame due to the foreign body reaction of the mesh, leading to fibrous granulation extending into the pores of the mesh. This framework prevents the recurrence with no tension in the posterior wall [6]. The mesh should essentially cover the potential spaces for recurrence. These include the direct hernia site, which is bounded by the rectus abdominis muscle medially, the inguinal ligament inferiorly, and the inferior epigastric vessels laterally. The mesh should also cover at least 3-5 cm beyond the deep inguinal ring (DIR) toward the anterior superior iliac spine (ASIS) to prevent a recurrence after the shrinkage of the mesh [7]. Anatomical knowledge and morphometric pattern in the particular population are essential to recommend the appropriate mesh size, which should cover the above-said potential sites for recurrence.

There are very few studies available in the literature on the correct size of the mesh to cover the said anatomical measures [8]. To the best of our knowledge, there are no such morphometric data available on the Indian population. Hence, this study was undertaken to study the morphometric features of the inguinal canal with the different types of inguinal hernias in the Indian population, to determine the appropriate size of the mesh required to cover the potential sites of recurrence.

## Materials and methods

The study was a prospective observational study, carried out in the department of anatomy in collaboration with the department of surgery over a period of three years. Institute Human Ethics Committee (IEC) approval was obtained for the study. The nature of, the methodology, and the risks involved in the study were explained to the patient and informed consent was obtained. All the information collected was kept confidential, and the patient was given full freedom to withdraw at any point during the study. All provisions of the Declaration of Helsinki were followed in this study.

All consecutive patients undergoing open inguinal hernia repair under local/regional/general anesthesia in the department of general surgery over a period of three years were included in the study. Inguinal hernia repairs that were done in emergencies for complicated hernia (irreducible, obstructed, or strangulated hernia), hernias repaired by laparoscopy, recurrent inguinal hernias, age less than 18 years, and hernias occurring following groin dissection were excluded from the study.

The study was designed as a prospective observational study, involving one group of patients. The sampling population included all patients admitted in the department of general surgery for elective open inguinal hernia repair under general/regional/local anesthesia over a period of three years, who were selected by a convenient sampling technique.

Patients with direct/indirect inguinal hernias planned for surgery were recruited for the study after assessing the inclusion/exclusion criteria. A detailed clinical examination was carried out for all patients and the details of the operating procedures were recorded such as duration of the procedure and type of surgery. The intraoperative parameters were studied in the patients, to predict the appropriate mesh size, including the position of the superficial inguinal ring (SIR) with its diameter, the position of DIR with its diameter, the location of DIR from ASIS, the location of SIR from ASIS, and the distance from the summit of the muscular arch to inguinal ligament. Measurements were taken by using a sterile divider and a metal scale under aseptic precaution.

Statistical analysis

Statistical analysis was done using SPSS 19.0 software version for Windows (IBM, Armonk, NY, US). Categorical variables, such as the distance of the internal oblique, the condition of the transversalis fascia, and the location of DIR from ASIS were summarised as frequency and proportion. All continuous variables, such as SIR and DIR with diameter and distance from the summit of the muscular arch to the inguinal ligament, were summarised as mean and standard deviation if data follow the normal distribution. The difference in morphometric details between the types of hernias was tested using the chi-square test. Categorical variables were assessed using the chi-square test. A p-value of less than 0.05 was considered statistically significant.

## Results

**Figure 1:**
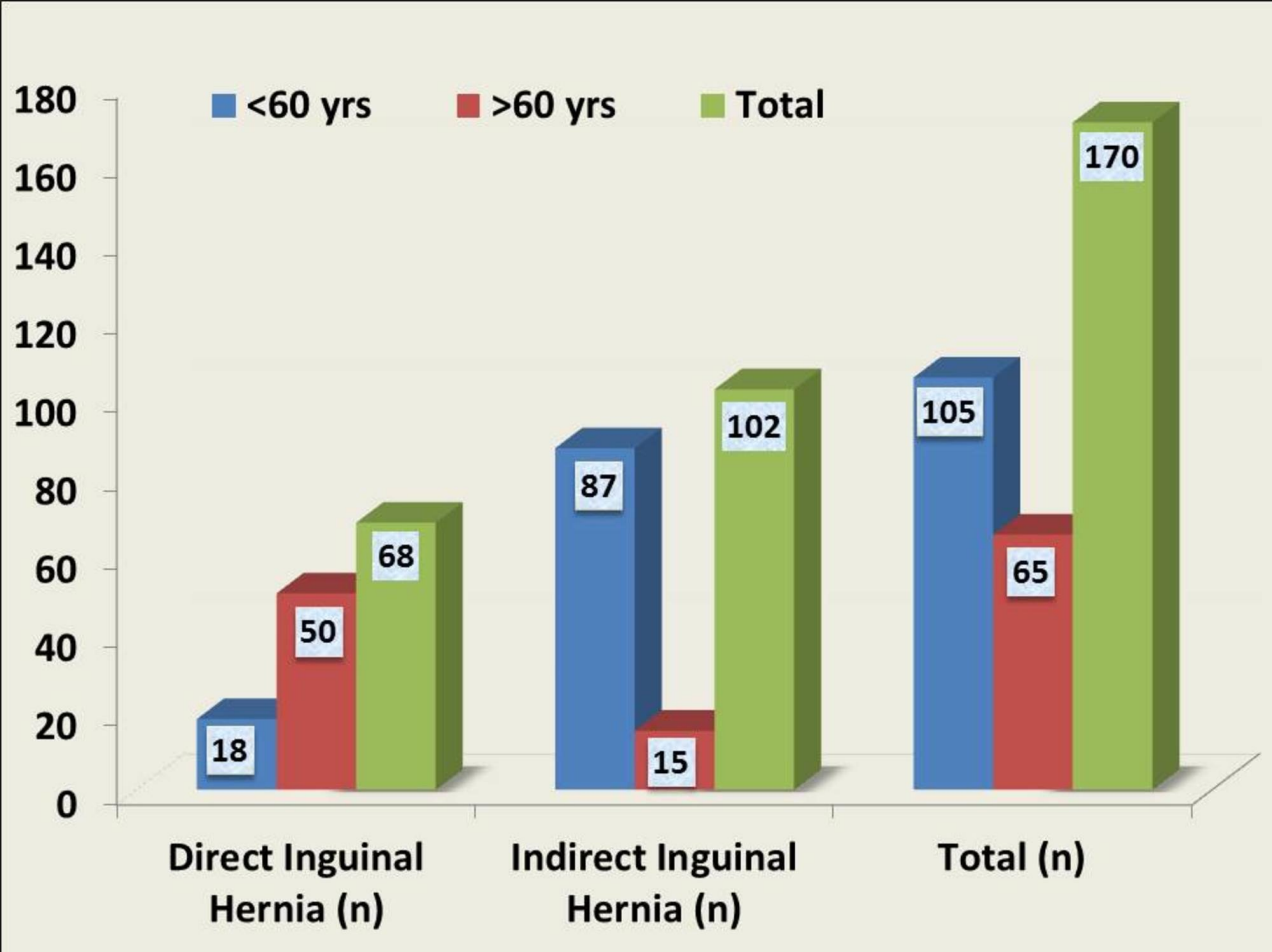
Comparison of types of hernias among different age groups in the study population yrs: years; n: number

The study included a total of 170 patients with ages ranging from 18 years to 91 years, with a mean age of 50.67 ± 17.59 years. A majority of the study population were male patients (n=167; 98.4%). Considering the type of inguinal hernia, the indirect inguinal hernia was the most common type with a frequency of nearly 60% (n=105). On comparing the type of hernia in both genders, the indirect hernia was the common type of hernia in both men and women in this study population. Only three patients were female in this study population and all three of them had an indirect inguinal hernia. An indirect hernia was the most common type in patients less than 60 years, comprising 82.7% (n=87). On analysis using the chi-square test, the difference was statistically significant (p=0.0001). A majority of the older group patients (n=50), aged more than 60 years, had a direct type of hernia (Figure 1).

**Figure 2:**
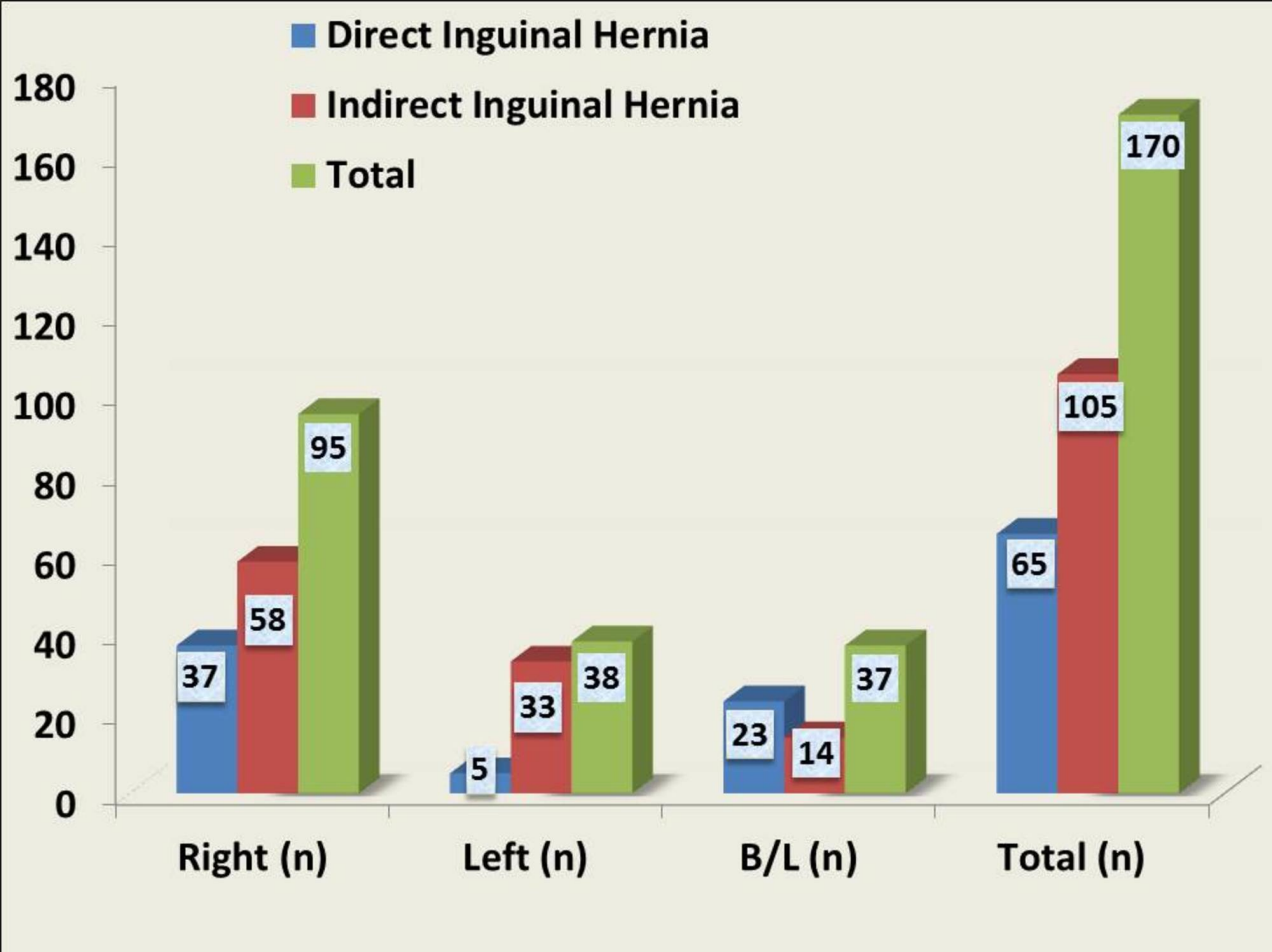
Comparison of types of hernias with side of occurrence in the study population B/L: bilateral; n: number

Of all the patients, 55.88% (n=95) had presented with a right-sided inguinal hernia. 22.35% patients (n=38) had left inguinal hernia and 21.76% patients (n=37) had bilateral hernia. In both the right and left sides, the common type was the indirect hernia. However, two-thirds of the bilateral hernias (n=23) were the direct type hernias. A direct hernia occurred mainly on the right side and bilaterally. Two-thirds of the patients (n=58) who had an indirect hernia were on the right side. A statistical analysis revealed a significant association of indirect hernias with a right-sided preponderance (Figure 2).

Distance from ASIS to SIR

The mean distance from ASIS to SIR in the study population was 10.2± 1.9 cm. The distance ranged from 5 cm to 20 cm. A statistical analysis showed a significantly (p=0.042) increased distance between ASIS to SIR in the indirect hernia patients compared to the direct hernia patients (Table 1).

**Table 1:** Comparison of distance from anterior superior iliac spine to superficial inguinal ring between direct and indirect hernias in the study population No: number of patients; SD: standard deviation; CI: confidence interval; ASIS: anterior superior iliac spine; SIR: superficial inguinal ring

Type of hernia (No.)	Distance from ASIS to SIR (cm)		p-value
	Mean	SD (95% CI)	
Direct hernia (n=68)	9.82	1.62 (9.45-10.18)	p=0.042
Indirect hernia (n=102)	10.58	2.02 (10.20-10.95)	

Distance from ASIS to DIR

The mean distance from ASIS to DIR in the study population was 4.14 ± 1.57 cm. The distance ranged from 1 cm to 10 cm. A statistical analysis showed a significantly (p=0.029) smaller distance between ASIS to DIR in the indirect hernia patients compared to the direct hernia patients (Table 2).

**Table 2:** Comparison of distance from anterior superior iliac spine to deep inguinal ring between direct and indirect hernias in the study population No: number of patients; SD: standard deviation; CI: confidence interval; ASIS: anterior superior iliac spine; DIR: deep inguinal ring

Type of hernia (No.)	Distance from ASIS to DIR (cm)		p-value
	Mean	SD (95% CI)	
Direct hernia (n=68)	4.16	1.57 (3.81-4.52)	p=0.029
Indirect hernia (n=102)	4.12	1.25 (3.88-4.35)	

Inguinal canal

The mean length of the inguinal canal in a direct hernia was 5.66 ± 0.5 cm whereas the mean length in an indirect inguinal hernia was 6.46 ± 0.8 cm. The increase in the length of the inguinal canal in an indirect hernia was statistically significant (p=0.029) (Table 3).

**Table 3:** Comparison of length of inguinal canal between direct and indirect hernias in the study population No: number of patients; SD: standard deviation; CI: confidence interval

Type of hernia (No.)	Length of inguinal canal (cm)		p-value
	Mean	SD (95% CI)	
Direct hernia (n=68)	5.66	0.5 (5.16-6.16)	p=0.029
Indirect hernia (n=102)	6.46	0.8 (5.66-7.26)	

SIR

The mean diameter of the SIR in the study population was 2.36 cm. The diameter ranged from 0.9 cm to 4 cm. A statistical analysis showed no significant difference in the diameter of the SIR in the indirect hernia patients compared to the direct hernia patients.

DIR

The mean diameter of the DIR in the study population was 2.36 cm. The diameter ranged from 0.9 cm to 4 cm. A statistical analysis showed no significant difference in the diameter of the DIR in the indirect hernia patients compared to the direct hernia patients.

Distance from the inguinal ligament to the summit of the muscular arch

The mean distance from the midpoint of the inguinal ligament to the summit of the muscular arch (conjoint tendon) in the study population was 4.03 cm. The diameter ranged from 0.5 cm to 6.5 cm. A statistical analysis showed no significant difference (p=0.358) in the distance from the inguinal ligament to the summit of the muscular arch in the indirect hernia patients compared to the direct hernia patients (Table 4).

**Table 4:** Comparison of distance from inguinal ligament to summit of muscular arch between direct and indirect hernias in the study population - complete hernia No: Number of patients; SD: Standard deviation; CI: Confidence interval

Type of hernia (No.)	Distance from inguinal ligament to summit of muscular arch (cm)		
	Mean	SD (95% CI)	p-value
Direct hernia (n=68)	3.36	1.22 (3.08-3.63)	p=0.358
Indirect hernia (n=102)	4.70	1.34 (4.45-4.95)	

## Discussion

An inguinal hernia is the most commonly performed elective operation in the general surgery operating room and is one of the surgeries where precise anatomical knowledge is of paramount importance to perfect the surgical skills and technique. Also, the accurate morphometric pattern in the particular population is essential to recommend the appropriate mesh size. A majority of the morphometric studies being cadaveric studies, only a few studies are available in the literature, assessing the inguinal hernia patients to determine the mesh size [9]. This study was undertaken to determine the appropriate size of mesh required to cover the potential sites of recurrence.

In this study population, the mean distance from ASIS to SIR was 10.2 ± 1.9 cm and to DIR was 4.14 ± 1.57 cm. Burcharth et al. measured an 8.8 cm length between ASIS and DIR [10]. The measurements were taken preoperatively in the study could actually overestimate the length due to the redundant skin and abdominal skin fat. The distance from ASIS to SIR and from ASIS to DIR was significantly longer in the indirect hernias in our study population. Direct hernias destroy the posterior wall, leading to enlarged SIR, thus causing the reduced length of the distance of ASIS from SIR. This fact is reflected in terms of a significantly reduced length of the inguinal canal in patients with direct hernias compared to indirect hernias.

The length of the inguinal canal, which was measured from DIR to SIR derived from subtracting the distance of ASIS to DIR from ASIS to SIR. Tanyel et al. studied the inguinal canal in the childhood population and concluded that the length remains short up to two years and increases proportionately to the age after two years in boys [11]. However, in girls, the length does not strictly increase with age. Burcharth et al. studied the inguinal canal in a group of prepubescent patients who developed hernias and reported that the length did not significantly vary depending on the side or gender [12]. The mean length of the inguinal canal in direct hernia patients in the present study was 5.66 ± 0.5 cm (CI: 5.16-6.16), which was significantly less than the mean length in indirect inguinal hernias (6.46±0.8 cm (CI: 5.66-7.26)).

The diameter of the DIR in hernia patients may be altered due to the content coming out through the DIR. Rabe et al. measured the DIR diameter as 2.2 ± 0.7 cm [9,12]. In the present study, the DIR diameter in indirect hernias was 1.78 ± 0.44 cm and in direct hernias, it was 1.86±0.46 cm, which was less as compared to previous studies. A majority of the indirect inguinal hernias in this study population had incomplete hernias, which dilate the DIR less severely than complete hernias. In the present study, the DIR diameter in indirect hernias was 2.2 ± 0.49 cm and in direct hernias, it was 2.51 ± 0.5 cm, which was similar to the previous studies [9,12].

The mean distance from the midpoint of the inguinal ligament to the summit of the muscular arch in the study population was 4.03 ± 1.2 cm. The diameter ranged from 0.5 cm to 6.5 cm. Rabe et al. measured the mean distance from the midpoint of the inguinal ligament to the summit of the muscular arch was 4.0 ± 1.2 cm, which is similar to the present study [9,12]. A direct hernia destroys the posterior wall and, hence, might increase the distance from the midpoint of the inguinal ligament to the summit of the muscular arch. A statistical analysis showed no significant difference in the distance from the inguinal ligament to the summit of the muscular arch in the indirect hernia patients compared to direct hernia patients in the present study. A majority of the direct hernias in our study population were bubonocele with a smaller size, which could have contributed to the less severe destruction of the posterior wall. 

Irving Lichtenstein put forth the concept of tension-free repair and achieved a breakthrough by using the Marlex prosthesis in the anterior approach repair [6,13]. Mesh repair to strengthen the posterior wall has become the standard of care all around the world, as it precludes the possibility of hernia recurrence by avoiding tension tissue repair and covers all the potential sites of hernia recurrence. Presently, open inguinal hernia repair with tension-free meshplasty is the most commonly performed operative procedure for inguinal hernia patients.

Choosing the appropriate mesh depends on the size, type of filament (prolene, vicryl, dual type, etc.), weight (regular, light-weight, ultra-lightweight), and the size of the porosity (macro-porous or micro-porous) of the mesh. Among the above-said parameters, the size of the mesh is of paramount importance, as a smaller size mesh inadequately covers all the defects and potential hernia sites and, hence, are bound to produce recurrence. Most of the mesh placed intraoperatively shrinks from 20%-40%, depending on many factors. It is generally observed that the mesh with a smaller pore size is likely to shrink more in the postoperative period [6,8,13].

The usual size of the mesh commonly placed for inguinal hernia repair ranges from 3 x 3 inches to 3 x 6 inches (7.5 x 7.5 cm to 7.5 x 15 cm). The recommended coverage during mesh placement is: the mesh should cover 2 cm medial (beyond) the pubic tubercle, 3-4 cm above the Hesselbach triangle and a minimum of 5-6 cm lateral to the DIR. The placement of a larger mesh, on the other hand, leads to palpable stiffness over the abdominal wall, restricted movements, and paresthesia [8,13]. Hence, choosing the correct size of mesh size requires an accurate estimation of inguinal canal parameters in inguinal hernia patients of a particular race, gender, and region.

Wolloscheck et al. tried measuring the diameter of the myopectineal orifice (MPO) to recommend the appropriate size of the mesh [13]. In their cadaveric-based study, the MPO dimensions were 7.8 x 6.5 x 4.5 cm. The study concluded that the 10 x 8 cm mesh should be the minimum size to be used for the hernia repair. Simons et al., in the European Hernia Society guidelines on the treatment of inguinal hernias in adult patients, recommended that the mesh should cover 2 cm medial to (beyond) the pubic tubercle, 3-4 cm above the Hesselbach triangle, and a minimum of 5-6 cm lateral to DIR [14].

**Figure 3:**
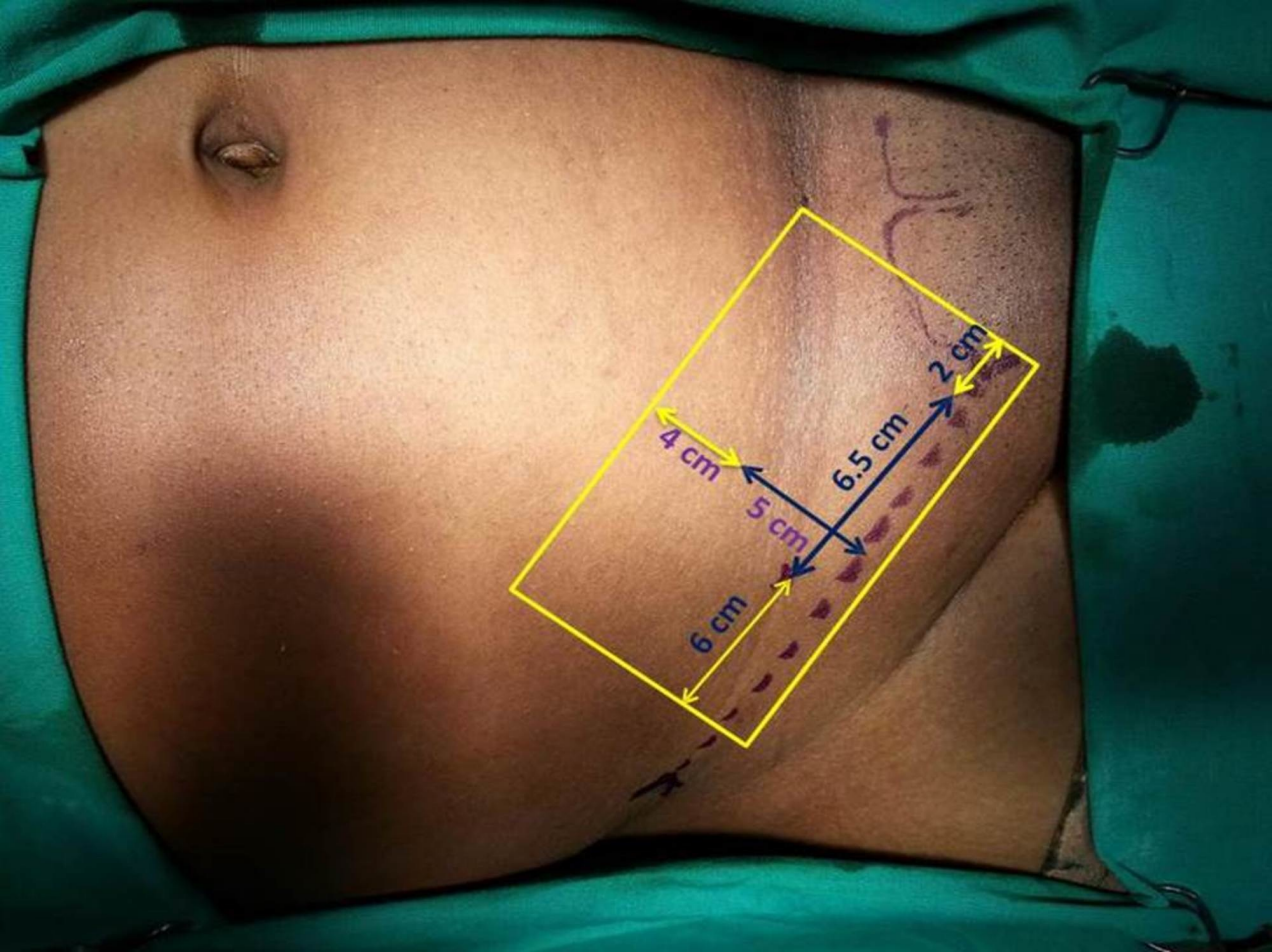
Appropriate mesh size based on morphometric measurements

Trabucco et al., in his attempt to measure the inguinal box to propose the appropriate mesh size, found the average distance of ASIS to DIR and DIR to pubic tubercle as 7 cm and 5 cm, respectively. The width extending from the inguinal ligament to the summit of the muscular arch averaged to 4.5 cm in his study [15]. Considering the above dimensions, Trabucco et al. suggested a mesh size of 8-9 cm to cover from the pubic tubercle to a point 3 cm beyond DIR [15]. Our study demonstrated the length of the inguinal canal (between SIR and DIR) to be 5.66-6.46. The mean distance from the midpoint of the inguinal ligament to the summit of the muscular arch in the study population was 4.03 ± 1.2 cm. The mean distance from ASIS to DIR was 4.14 ± 1.57 cm. The mean diameter of SIR was 2.5 cm and the mean diameter of DIR was 2 cm. Considering the above morphometric assessments and the European Hernia Society guidelines, the ideal mesh size in our population would be: transversely 15 cm (6 (required beyond the DIR) + 6.5 (length of the inguinal canal + 2 (required beyond the pubic tubercle)) and vertically 9 cm (5 (distance from the midpoint of the inguinal ligament to the summit of the muscular arch) + 4 (required above the Hesselbach triangle)); i.e., 15 x 9 cm mesh to cover all the potential sites of recurrence (Figure 3).

## Conclusions

Considering the morphometric assessments of the length of the inguinal canal (5.66 - 6.46), the mean distance from the midpoint of the inguinal ligament to the summit of the muscular arch (4.03 ± 1.2 cm), the mean distance from ASIS to DIR (4.14 ± 1.57 cm), and the recommendations of European Hernia Society guidelines regarding the minimum surface to be covered by mesh, the ideal mesh size for the population would be 9 x 15 cm to cover all the potential sites of recurrence.
